# Long-term health outcomes of adolescent character strength interventions: 3- to 4-year outcomes of three randomized controlled trials of the Shamiri program

**DOI:** 10.1186/s13063-022-06394-7

**Published:** 2022-05-25

**Authors:** Katherine E. Venturo-Conerly, Natalie E. Johnson, Tom L. Osborn, Eve S. Puffer, Thomas Rusch, David M. Ndetei, Christine M. Wasanga, Victoria Mutiso, Christine Musyimi, John R. Weisz

**Affiliations:** 1 Shamiri Institute, Nairobi, Kenya; 2 Shamiri Institute, Allston, MA USA; 3 Department of Psychology, Harvard University, Cambridge, MA USA; 4 Global Health Institute, Duke University, Durham, NC USA; 5 Competence Center for Empirical Research Methods, WU Vienna University of Economics and Business, Vienna, Austria; 6 African Mental Health Research & Training Foundation, Nairobi, Kenya; 7 Department of Psychiatry, University of Nairobi, Nairobi, Kenya; 8 Department of Psychology, Kenyatta University, Nairobi, Kenya

**Keywords:** Adolescents, sub-Saharan Africa, Global Mental Health, Depression, Anxiety, Wellbeing, Global Health, Shamiri, Randomized controlled trial

## Abstract

**Background:**

Adolescents in low- and middle-income countries in need of mental health care often do not receive it due to stigma, cost, and lack of mental health professionals. Culturally appropriate, brief, and low-cost interventions delivered by lay-providers can help overcome these barriers and appear effective at reducing symptoms of depression and anxiety until several months post-intervention. However, little is known about whether these interventions may have long-term effects on health, mental health, social, or academic outcomes.

**Methods:**

Three previous randomized controlled trials of the Shamiri intervention, a 4-week, group-delivered, lay-provider-led intervention, have been conducted in Kenyan high schools. Shamiri teaches positively focused intervention elements (i.e., growth mindset and strategies for growth, gratitude, and value affirmation) to target symptoms of depression and anxiety and to improve academic performance and social relationships, by fostering character strengths. In this long-term follow-up study, we will test whether these mental health, academic, social, and character-strength outcomes, along with related health outcomes (e.g., sleep quality, heart-rate variability and activity level measured via wearables, HIV risk behaviors, alcohol and substance use), differ between the intervention and control group at 3–4-year follow-up. For primary analyses (*N*_anticipated_ = 432), youths who participated in the three previous trials will be contacted again to assess whether outcomes at 3–4-year-follow-up differ for those in the Shamiri Intervention group compared to those in the study-skills active control group. Multi-level models will be used to model trajectories over time of primary outcomes and secondary outcomes that were collected in previous trials. For outcomes only collected at 3–4-year follow-up, tests of location difference (e.g., *t*-tests) will be used to assess group differences in metric outcomes and difference tests (e.g., odds ratios) will be used to assess differences in categorical outcomes. Finally, standardized effect sizes will be used to compare groups on all measures.

**Discussion:**

This follow-up study of participants from three randomized controlled trials of the Shamiri intervention will provide evidence bearing on the long-term and health and mental health effects of brief, lay-provider-delivered character strength interventions for youth in low- and middle-income countries.

**Trial registration:**

PACTR Trial ID: PACTR202201600200783. Approved on January 21, 2022.

**Supplementary Information:**

The online version contains supplementary material available at 10.1186/s13063-022-06394-7.

## Administrative information

Note: the numbers in curly brackets in this protocol refer to SPIRIT checklist item numbers. The order of the items has been modified to group similar items (see http://www.equator-network.org/reporting-guidelines/spirit-2013-statement-defining-standard-protocol-items-for-clinical-trials/).

**Table:** 

Title {1}	Long-term health outcomes of adolescent character strength interventions: three-to four-year outcomes of three randomized controlled trials of the Shamiri program
Trial registration {2a and 2b}.	Pan African Clinical Trials Registry: PACTR202201600200783
Protocol version {3}	1.0
Funding {4}	Primary funding for this trial was provided by the Templeton World Charity Foundation, grant #TWCF0633. Secondary funding for this trial was provided by Alchemy Pay.
Author details {5a}	^1^Shamiri Institute, Nairobi, Kenya & Allston, MA, USA ^2^Department of Psychology, Harvard University, Cambridge, MA, USA ^3^Global Health Institute, Duke University, Durham, NC, USA ^4^Competence Center for Empirical Research Methods, WU Vienna University of Economics and Business, Vienna, Austria ^5^African Mental Health Research & Training Foundation, Nairobi, Kenya ^6^Department of Psychiatry, University of Nairobi, Nairobi, Kenya ^7^Department of Psychology, Kenyatta University, Nairobi, Kenya
Name and contact information for the trial sponsor {5b}	Templeton World Charity Foundation Phone: 2423624904
Role of sponsor {5c}	This study was primarily funded by the Templeton World Charity Foundation, located at Bayside Executive Park, Bldg. #2, West Bay Street and Blake Rd., PO Box N-7776, Nassau, Bahamas. Secondary funding was received from Alchemy Pay, located at Novelty TechPoint 0502, 27 New Industrial Road, Singapore. The study design; collection, management, analysis, and interpretation of data; writing of the report; and the decision to submit the report for publication are the sole responsibilities of the authors.

## Introduction

### Background and rationale {6a}

Recently, there has been increasing recognition of mental health as an essential component of health, with the World Health Organization (WHO) declaring that health cannot exist without mental health [1]. Reports of mental health issues among children and adolescents are increasing globally, especially due to the COVID-19 pandemic [2], with around 20% of children and adolescents having a mental health condition, and most mental health disorders originating between the ages of 12 and 24 [3, 4]. Poor mental health is associated with many additional concerns for health and development among young people, including lower educational achievement, substance use problems, risk of experiencing and perpetrating violence, relationship problems, poor reproductive and sexual health, and suicide risk [4–8].

School-going adolescents in many parts of the world, particularly in low-and middle-income countries (LMICs) such as those in sub-Saharan Africa (SSA), are in urgent need of innovative ways to address mental health burdens, as they face several barriers to accessing care. Such barriers include a dearth of trained mental health clinicians and stigma associated with mental health conditions and care [9, 10]. Additionally, the duration and cost of most mental health treatments—many of which were not designed for use in SSA [11]—are prohibitive to many youths and families [4, 12].

These barriers to care have led researchers to test low-cost, low-stigma, and scalable mental health solutions in SSA, some of which could be considered character strength interventions (i.e., interventions to build character strengths and apply them to one’s life) [13] and wise interventions (i.e., brief, precise interventions targeting specific psychological processes that influence meaning-making about the self and the world) [14]. One intervention drawing from this literature, Shamiri, meaning “Thrive” in Kiswahili, is delivered in a group setting by near-peer high school graduate lay-providers, and is designed to change the way high-school-aged youths view themselves and the world [15, 16]. Shamiri takes four hours to implement over the course of four weeks, and has three components: growth mindset [17] and strategies for growth, gratitude [18], and value affirmation [19].

Previous research on Shamiri in Kenya has shown promising improvements in youth mental health, psychosocial, and academic outcomes at post-intervention and seven-months follow-up [17, 20]. In a randomized controlled trial (RCT) of Shamiri with 51 Kenyan adolescents with clinically elevated depression and/or anxiety symptoms, we found improvements in depression and anxiety symptoms, academic performance, and perceived social support from friends when compared to an active study skills control group immediately post-intervention [15]. In a well-powered (*N* = 413), preregistered replication of this study [16], youths in the Shamiri intervention exhibited improvements in depression and anxiety symptoms over time, and when compared to the active study skills control group. These improvements were maintained at 7 months post-intervention [20]. This study also indicated that Shamiri is feasible and acceptable to Kenyan youths. A third ongoing trial is intended to investigate the effects of each of the three components of the Shamiri intervention against the full Shamiri protocol and an active study skills control group [21].

Our previous work which focused on developing and testing Shamiri suggests that this short character strength intervention has the potential to improve social, academic, and mental health outcomes of youth in Kenya. An important next step is to evaluate Shamiri’s potential for improving these and other related health outcomes in the long-term. Therefore, in this proposed study, we will investigate the Shamiri intervention’s effects, relative to the active control condition’s effects, on a wider variety of health, wellbeing, and social outcomes at 3–4-year follow-up of participants in our three prior RCTs. To the best of our knowledge, this will be the first study to test the effects of character strength interventions on such a rich range of health outcomes over such an extended period.

### Objectives {7}

This study has four goals. Our primary goal is to compare the effects of the Shamiri intervention and control condition on the primary outcomes (depressive symptoms, anxiety symptoms, academic performance, and social support) in the long-term. We hypothesize that the intervention group will outperform the active control on each of our primary outcomes. Secondarily, we aim to compare the effects of each group on secondary outcomes (described below in {12}). We also aim to explore for whom these interventions are most effective in the long-term. Finally, in an exploratory fashion, we wish to compare effects for the intervention groups that received a single component of the Shamiri intervention against the group that received the full Shamiri intervention and the active control group.

### Trial design {8}

This study is a parallel-group randomized comparative effectiveness trial with five arms: (1) the Combined Shamiri Intervention (consisting of a growth element, a gratitude element, and a value affirmation element), (2) the Growth Intervention, which encompasses content related to growth mindset and strategies for growth, (3) the Gratitude Intervention, which incorporates content to build feelings of gratitude and expression of gratitude, (4) the Values Intervention, which incorporates content to help students identify, affirm, and better act on their personal values and sense of purpose, and (5) a Study Skills Control, an active control group teaching students study skills that may be useful to them as students. Each condition is described further in section {11a}. All participants in this trial have already completed intervention in these conditions in three previous RCTs. The first and second RCT had two conditions, the Combined Shamiri Intervention, and the Study Skills control, and these interventions were administered with a 1:1 ratio, or equal probability for assignment to each of the two conditions. In the third RCT, participants were allocated within each school to the five conditions with a 1:1:1:1:1 ratio or equal probability for assignment to each of the five conditions. Each arm required 4-h-long sessions spanning 4 weeks and was delivered in a group of 8–15 students by a local lay provider, aged between 18 and 24. Measures of mental health, wellbeing, and character strengths were collected in the three previous RCTs at baseline, 2-week midpoint, 4-week endpoint, and 1-month, 3-month, 7-month, and 9-month follow-up. For this long-term follow-up, measures of mental health, wellbeing, character strengths, stress, health behaviors, socioeconomic and health status will be collected at 3–4 years follow-up. Academic data was collected in the three previous RCTs for the school terms before, during, and immediately after the intervention. For the current trial, academic data will be collected for the last year of secondary school. De-identified trial data will be available upon request.

## Methods: participants, interventions, and outcomes

### Study setting {9}

This study will be conducted with participants who attended secondary school in Kiambu and Nairobi counties in Kenya. For participants under 18 or still in school, we will visit their schools to conduct data collection. Data collection for adults no longer in school will take place in the Nairobi area at the African Mental Health Research and Training Foundation (AMHRTF) offices. Adult participants will present themselves to AMHRTF for self-reports and assessment by a clinician. These participants will also visit Kenyatta National Hospital’s Voluntary Counselling & Testing Centre if they wish to know their HIV status.

### Eligibility criteria {10}

All participants who have taken part in three previous RCTs [15, 20, 21] of the in-person, group-based, 4-week Shamiri intervention, and had elevated symptoms of depression or anxiety at baseline of the previous RCTs (defined as scores of 10 or over on the Generalized Anxiety Disorder-7 (GAD) and 15 or over on the Patient Health Questionnaire-8 (PHQ-8)) are eligible for enrolment in this long-term follow-up study. Participant consent or assent, and in the case of minors, guardian consent, will also be required for participation. No additional exclusion criteria will be applied.

### Who will take informed consent? {26a}

A member of the study team who is from and living in Kenya will seek informed consent and assent prior to collecting follow-up measures. We will provide participants with a digital consent form (see supplementary materials for the model consent form to be used). Then, a member of the study team with appropriate ethical training and Institutional Review Board (IRB) approval will walk the participants through the consent form and clarify any questions they may have. They will emphasize the voluntary nature of participation in the study.

While we hope to seek permission/consent from parents of minors who wish to participate in our study, following local customs of boarding schools in Kenya, where students do not have direct access to their parents, as well as the logistical difficulty in obtaining written consent from parents disbursed throughout the country with limited access to timely postal communication, we will work with the school administration to obtain consent from the parents of minors. Specifically, we will work with school administrators to obtain informed consent on behalf of parents using the customary methods of the school (i.e., sending text messages or calling parents). The participants who are minors will also be asked to provide informed assent on site.

### Additional consent provisions for collection and use of participant data and biological specimens {26b}

We will seek additional consent prior to taking physical measures of health from participants. They will be informed that these measures are voluntary and that they may opt out of them at any time as they wish. We will also seek additional consent from participants over 18 years old who are not in school and wish to attend Kenyatta National Hospital’s Voluntary Counselling & Testing Center for an HIV test and inform participants that if they wish to be tested for HIV, we will pay for the test, and if they wish, they may report the result of the test back to the study team, but that they are not required to do so.

This trial does not involve collection, storage, or analysis of biological specimens.

### Interventions

#### Explanation for the choice of comparators {6b}

For primary analyses, the Shamiri intervention will be compared to a Study Skills active control condition of equal duration and highly similar format. As detailed further in a protocol for one of the three RCTs [21] from which participants will be drawn for this study, the Study Skills active control condition was selected as a comparator for several reasons. First, active control conditions typically provide a more robust comparison than no-treatment or waitlist control conditions [22]. Second, because students in Kenyan high schools report pressure to succeed academically at high rate and severity [23], improving study skills may be practically useful and may help reduce stress and anxiety for students. Third, past research has shown that symptoms of mental health problems may indeed be somewhat alleviated by the Study Skills control condition [15]. Finally, those in the Study Skills control condition participated in weekly small group sessions led by a lay-provider; this regular contact may have helped lower barriers to requesting needed support from a lay-provider.

#### Intervention description {11a}

Participants in three RCTs have previously completed their participation in the intervention and control conditions. Each condition consisted of four sessions, lasting 1 h per session for a duration of 4 weeks. The intervention and control conditions were delivered by a near-peer lay provider to groups of approximately 8–15 students. Participants will not participate in the intervention or control condition again as part of this study but instead will complete follow-up measures to assess the long-term effects of these interventions. These interventions are further detailed in the intervention descriptions of our previous three RCTs [15, 20, 21]. Each condition consisted of an equal dose of total intervention time: four 1-h weekly sessions that focused on teaching and reinforcing the knowledge and application of the active ingredient of the condition. Each single-element intervention (i.e., growth only, gratitude only, or values only intervention) included the elements related to this one element in the combined Shamiri condition, along with additional related activities to teach and practice the same principles. Sample intervention protocols can be found in the supplementary materials for a previous article on Shamiri [21]. The general format for each intervention was as follows:

- In session 1, participants completed baseline measures, were introduced to the guidelines for the group and completed group activities to introduce themselves. They learned through educational discussions and simple, fun activities about the scientific literature of their assigned intervention and how the intervention might help them in their life. Session 1 typically ended with a group discussion about the active ingredient.
- In session 2, participants heard stories and anecdotes related to the active ingredient of their assigned condition. Participants also completed a writing exercise to digest information from this session. Mid-point measures were collected at the end of session 2.
- In session 3, participants completed evidence-based exercises related to the active ingredient which taught participants how to apply what they have learned to solve real-life problems. Participants were also typically asked to apply these techniques to a hypothetical scenario.
- Session 4 summarized what participants had learned about the active ingredient through discussion and group activities. Participants completed endpoint measures at the end of this session. The active ingredients which participants were previously randomized to are described below:

#### Combined Shamiri intervention

Participants who received this condition completed modules related to the active ingredients described below: growth, gratitude, and values. The first two sessions focused on growth mindset, the third session focused on gratitude, and the final session focused on values. These sessions incorporated a mixture of lecture, discussion, and group exercises.

#### Gratitude intervention only

Some participants received only the gratitude intervention, which taught them how to intentionally notice, communicate, and appreciate feelings of gratitude. Participants also learned how to practice gratitude on a daily basis in their lives. Group leaders emphasized the importance of verbalizing and consciously thinking about what we are grateful for, as well as the benefits this has on wellbeing. Group exercises included how to better express gratitude and incorporate it into everyday life.

#### Values intervention only

In this condition, participants learned about identifying and living according to their core values. Participants shared their understanding of values with their group and the values that were most relevant to their lives. Participants then chose personal values and wrote about how they have demonstrated those values. They completed activities such as considering how they could live more in accordance with their values, telling stories of inspiring role models, and making goals for applying their values to their lives.

#### Growth intervention only

Participants who received the growth condition only learned about neuroplasticity, which is the ability of the brain to grow and change over time and with effort. In groups, participants completed activities such as a saying-is-believing exercise, wrote personal growth stories, heard testimonials, and discussed and considered how to apply strategies for solving problems and growing.

#### Study skills control

This control condition was developed in collaboration with Kenyan experts in research and education. Participants who received this condition learned skills designed to improve abilities to study and ultimately academic performance. As part of this condition, participants learned tips related to skills such as note taking, critical reading, and essay writing, as well as how to implement a “study cycle:” studying throughout the year instead of cramming right before exams.

### Criteria for discontinuing or modifying allocated interventions {11b}

Because no interventions will be delivered during this trial, there are no criteria for discontinuing or modifying interventions. As for the outcome measurement protocol, participants will be informed that they may refuse to answer any questions asked as part of outcome measures collected by the study team and that they may withdraw their consent to participate in the study at any time.

### Strategies to improve adherence to interventions {11c}

Because no interventions will be delivered during this trial, no strategies for improving adherence to interventions are necessary. As for the outcome measurement protocol, adherence to that will be ensured via extensive training and monitoring of outcome assessors, as detailed under Data Collection and Management below.

### Relevant concomitant care permitted or prohibited during the trial {11d}

Participants were not prevented from seeking care outside of the study during the 3–4 years since participating in the Shamiri intervention trials. However, because of a paucity of trained mental health care providers [9] and elevated stigma associated with mental illness and mental health care in Kenya [10], it is unlikely that many participants in the Shamiri trials have received formal mental health care in the years since participating in the Shamiri intervention trials.

### Provisions for post-trial care {30}

We do not anticipate harm caused by participating in this study, and thus have no plans for compensation in case of harm. For those participants who present with elevated risk of harm to self or others, or who report need for continued support or care for other reasons (e.g., substance use, abuse, need for essential resources such as food), the study team and primary care clinician will collaboratively implement an emergency protocol (located in supplementary materials) and will provide all adult participants who have completed school with a resource sheet including relevant local resources. For participants who are still minors and/or in school, the study team and primary care clinician will collaborate with guardians including school officials to provide necessary resources and care according to local customs.

### Outcomes {12}

As part of the three RCTs from which we will recruit participants for this trial, many of the outcomes below have been previously collected from participants. In each measure below, it is indicated whether it was collected in previous trials. All outcomes below will be collected from participants in this RCT once, at 3–4 years follow-up.

For full details of planned analyses, see {20} below. In addition to these analyses, summary statistics of central tendency (e.g., averages) and measures of dispersion/variability (e.g., standard deviations), and when relevant, 95% confidence intervals for scores of each outcome measure will be calculated and reported.

### Primary outcome measures

#### Patient Health Questionnaire-8

The PHQ-8 will be used to assess the severity of symptoms of depression [24, 25]. PHQ-8 is an 8-item version of the PHQ-9 that excludes the final item which asks about suicidal ideation. Local Kenyan experts, including researchers and school officials, have instructed us to exclude this item due to the stigma associated with suicidal ideation, which could be harmful and off-putting to participating and potential schools and students. PHQ-8 scores have been shown to be highly correlated with PHQ-9 scores; hence, the same scores can be used to determine severity of depression [24, 26]. The PHQ-8 has been used to classify depression among Kenyan adolescents [20, 27, 28], and has been measured among all participants who could be recruited from the three previous trials of Shamiri. It has been validated for use in this population [15, 20, 21, 29].

#### Generalized Anxiety Disorder Screener-7

The GAD-7 will be applied to assess for symptoms of anxiety. This tool is used worldwide to screen for anxiety in adult and adolescent populations [30] and has been used to study anxiety symptoms of Kenyan adolescents [17, 27, 28]. This instrument has been used to measure anxiety symptoms of the participants in all three previous Shamiri RCTs from which this sample will be drawn [15, 20, 21]. It has also been validated for use in this population [29].

#### Academic performance

Academic grades of participants will be collected for the first term, concluding usually between April and July, and the second term, which usually concludes between September and November, of the year in which follow-up data is collected (or the final year of school for those who have graduated). Scores on the Kenya Certificate of Secondary Education (KCSE) examination will be collected for those participants who have taken the exam prior to the conclusion of the study. For participants who will not take the KCSE by the conclusion of the study, scores will be collected for the KCSE mock examinations in the year prior to the examination.

We will calculate the average grade for each student for term 1 and term 2 across all enrolled subjects to determine their academic performance. Although the number of subjects per student differs across schools, students typically sign up for between 6 and 12 subjects. To compare grades across different schools, grade levels, and academic subjects, we will use the standardization method from our pilot RCT [15]. We will convert academic grades to standard scores with a mean of 60 and standard deviation of 10, chosen arbitrarily and used in rescaling. We may also examine discrete averages for subject-specific, math and science, or humanities scores in the same fashion. The grades of the participants in this trial have been collected in all three previous RCTs of Shamiri [15, 20, 21].

#### Multidimensional Scale of Perceived Social Support–8

We will use the Multidimensional Scale of Perceived Social Support (MSPSS) to gauge personal satisfaction with social support [31]. There are three subscales: the friends subscale measures support from friends, the family subscale measures support from family, and the significant others subscale measures support from significant others. The MSPSS has been used with Kenyan adolescents, and shows adequate internal consistency [27]. This measure has been collected previously from all participants in this trial as part of all three previous Shamiri trials, and has shown adequate psychometric properties [15, 20, 21].

### Secondary outcome measures

#### Short Warwick-Edinburgh Mental Well-being Scale (SWEMBS)–7

The Short Warwick-Edinburgh Mental Wellbeing Scale (SWEMBS) will be used to determine subjective wellbeing of participants [32]. The SWEMBS (7-item) is a shortened version of the Warwick-Edinburgh Mental Wellbeing Scale (WEMWBS), a 14-item tool developed for public mental health monitoring and assessment, which encompasses the promotion of mental wellbeing, as well as the prevention of and recovery from mental illness [33]. The short version is used extensively in large-scale psychological surveys and interventions due to its lower burden on participants [32, 34–36]. The SWEMBS has been used previously with South African youths [37]. Some of the participants in this RCT have completed the SWEMBS measure in the ongoing RCT of Shamiri [21].

#### Engagement, Perseverance, Optimism, Connectedness, Happiness Measure of Adolescent Wellbeing

We will use the Engagement, Perseverance, Optimism, Connectedness, Happiness Measure of Adolescent Wellbeing (EPOCH) to assess positive psychological characteristics that could foster wellbeing, physical health, and other positive outcomes in adulthood. EPOCH is a 20-item measure that has been validated for use with adolescents in the United States and Australia [38]. Some of the participants in this trial have been measured on the optimism and happiness subscales in a previous Shamiri RCT [16], and other participants have been measured on all five of the subscales in our ongoing RCT [21].

#### Purpose in Life Scale–12

The Purpose in Life Scale (PILS) will be used by our team to measure sense of purpose. The PILS is a 12-item scale that has been widely used for research and screening purposes, and demonstrates strong psychometric validity [39]. This measure has been used for some of the participants in this trial as part of an ongoing RCT of Shamiri [21].

#### Gratitude Questionnaire–6

To determine experience of gratitude, we will use the Gratitude Questionnaire (GQ-6), a 6-item self-report questionnaire. This instrument has been established to measure grateful disposition—the characteristic of recognizing positive experiences, valuable support and actions from others in everyday life [18, 40]. This questionnaire has been shown to have strong psychometric validity among adolescents [41] and has been used in RCTs with Kenyan adolescents [20, 28]. This measure was collected from some of the participants in this trial as part of the two most recent RCTs of Shamiri [16, 21].

#### Perceived Control Scale for Children

The Perceived Control Scale for Children (PCSC) measures the perception that one can attain desired outcomes and avoid undesired outcomes through effort [42]. When employed with Kenyan adolescents, this scale demonstrated adequate internal consistency [27]. This measure has been used for these participants in the three previous trials of Shamiri [15, 20, 21].

#### Secondary Control Scale for Children

The Secondary Control Scale for Children (SCSC) is used to determine children’s perceived ability to exert secondary control [43]. It measures to what extent children perceive their influence on the psychological impact of objective conditions. This scale has demonstrated adequate validity and reliability among a large sample of North American youths. Some of the participants in this trial were administered the SCSC as part of the ongoing RCT of Shamiri [21].

#### Adolescent Sleep-Wake Scale

Quality of sleep will be assessed by a clinician, using the Adolescent Sleep-Wake Scale (ASWS), which has been validated for use with adolescents and young adults [44]. Quality of sleep has been shown to correlate with physical and mental health among adolescents [45]. The ASWS is a 28-item measure which includes five subscales: going to bed, falling asleep, maintaining sleep, reinitiating sleep, and return to wakefulness. Total and subscale scores are an average of scores on each item, with answers ranging from 1 (“always”) to 6 (“never”), with higher scores indicating better sleep quality. The ASWS has been validated for use with adolescents, with internal consistency as measured by Cronbach’s alpha between 0.64 and 0.82 [46].

#### Alcohol Use Disorders Identification Test

The Alcohol Use Disorders Identification Test (AUDIT) is a 10-item measure used to detect hazardous or harmful alcohol consumption. It has three domains: alcohol consumption, drinking behavior, and alcohol-related problems [47]. A primary care clinician will administer this test, and we will slightly modify the WHO-recommended cutoff scores to use culturally appropriate cutoff scores previously identified among a Kenyan sample, with normal use indicated as a score between 4 and 12, harmful use between 13 and 18, and alcohol dependence registered with a score of 19 or greater [48, 49].

#### Alcohol Smoking and Substance Involvement Screening Test

For participants who are 18 and over, substance use will be measured by a clinician using WHO’s Alcohol, Smoking and Substance Involvement Screening Test (ASSIST). Participants will be asked about their use of alcohol, tobacco, and other drugs within the 3 months prior to interview. This test has been piloted successfully for use with adolescents and adults [50] and will be modified as indicated by local advisors for the purposes of this study to suit the local context.

#### Pregnancy and children

A primary care clinician will interview participants 18 and over who are not attending school about pregnancy and children. The clinician will use this questionnaire to determine whether the participant or their partner is currently pregnant or has been pregnant since participation in the Shamiri program, whether they have children, and whether the pregnancy and/or child were expected. This questionnaire was developed based on a tool used in a study of youth sexual harassment [51].

#### Violence Against Women Instrument

A clinician will interview participants 18 and over who are not attending school about intimate partner violence (IPV) using WHO’s 21-item Violence Against Women Instrument (VAWI) [52]. This tool has been formulated to measure victimization and perpetration of violence among men and women [53]. For the purposes of this study, we will use a 20-item version of the VAWI which has been used previously to measure IPV in SSA [54]. VAWI demonstrates adequate internal consistency to measure both victimization and perpetration among youths in SSA [54], and has been used in Kenya to measure IPV among adolescent girls and young women [55].

#### HIV status and risk behavior

Participants who are 18 and over will be interviewed by a clinician regarding HIV risk behaviors, such as number of sexual partners and condom use. This questionnaire is based on studies of HIV risk in Kenyan and Ethiopian youths [56, 57]. Participants will then be asked if they are aware of their HIV status and may volunteer to report their status to the clinician if known. If their status is unknown, adult participants who are not in school will be invited to take a free HIV test at Kenyatta National Hospital’s Voluntary Counselling & Testing Centre. Participants will also be informed that this part of the study is completely voluntary and that upon receiving the result, they may report the result to the study team if they wish. If they choose to take the voluntary HIV test, they will also receive pre- and post-test counseling at the testing center.

#### Heart rate variability

A research assistant will administer a smart watch to participants and instruct them to wear the watch for two weeks in order to measure pulse. From this measure, pulse rate variability will be calculated, which is highly correlated with heart rate variability (HRV) [58]. HRV is the variance in timing between two heart beats and has been shown to approximate psychological health and stress levels [59]. Previous studies have used smart watches to measure HRV of children [60], and have shown that HRV is correlated with anxiety [61]. Smart watches are low-risk and non-invasive and have been shown to be acceptable for everyday wear [62] and feasible for health informatics use [63].

#### Physical activity

Smart watches will also be used to measure activity level of participants. This will be determined by using the number of steps and overall distance moved in a period of 2 weeks. A previous study used smart watches to measure physical activity of participants, and it was shown to correlate with depressive symptoms [61].

#### History of hospitalization

History of hospitalization will be collected through interview with a clinician, who will ask participants about their hospitalization history since participating in the study. Data points include whether the participant has been hospitalized at all post-intervention, the number of times the participant has been hospitalized post-intervention, whether the participant was admitted overnight, and the reason for hospitalization. Previous studies have shown a relationship between history of hospitalization and mental health, premature/unplanned pregnancy, HIV status, and other public health outcomes [64–66].

#### Anthropometric and health measures

The clinician will take several physical measures to assess the general health of participants. Blood pressure will be taken using a blood pressure cuff. Weight and height of participants will be taken in order to calculate body mass index (BMI). Waist and hip circumference will be taken with a tape measurer to calculate the waist-to-hip ratio. Participants will also be asked if they are currently taking any prescription medications or if they have been prescribed any medications in the past year.

#### Demographic questionnaire

The clinician or research assistant will ask participants to provide basic demographics including age and gender, as well as socio-demographics including employment and education status, and living situation. Age and gender have been measured for these participants in our previous three trials [15, 20, 21].

#### Household wealth

A clinician will administer a survey of household wealth based on current living situation to approximate the socioeconomic status of participants. The measure we will use was developed as part of a recent study with Kenyan adolescents. (unpublished data)

#### Participant timeline {13}

The participants in this study have already completed interventions as part of three previous RCTs testing Shamiri [15, 20, 21]. As part of the current RCT, participants will be contacted again for long-term follow-up. Per Table 1, participants from the first cohort completed the intervention in June 2018 and will be contacted for informed consent in January 2022. Immediately following consent, these participants will complete a battery of long-term follow-up measures, to include primary measures which have been collected as part of the previous trial, secondary self-report measures, some of which were collected in this trial, and additional health and behavioral measures which have not been collected before. Participants in the second cohort completed the intervention in July and August 2019 and will be contacted to provide informed consent to participate in this study in September and October 2022. Immediately following consent, participants will complete all follow-up measures as part of this study in September and October 2022. Participants who are part of the third RCT completed the intervention between May and July 2021. These participants will be contacted for consent to participate in this study between August and November 2024 and will complete follow-up measures which are part of this study immediately following their consent.

**Table 1:** Schedule of previous intervention implementation and study follow-up outcome measures

Time point	STUDY PERIOD												
	Enrolment			Allocation			Post-allocation						Close-out
	Jun 2018	Jun-Jul 2019	May 2021	Jun 2018	Jul 2019	May 2021	Jun 2018	Jul-Aug 2019	May-Jul 2021	Jan 2022	Sep-Oct 2022	Aug-Oct 2024	Nov 2024
**ENROLMENT**^**a**^													
Eligibility screen	x	x	x										
Informed consent	x	x	x							x	x	x	x
Allocation				x	x	x							
**INTERVENTIONS**^**a**^													
Combined Shamiri							x	x	x				
Growth									x				
Gratitude									x				
Values									x				
Study skills control							x	x	x				
**ASSESSMENTS**^**b**^													
Demographic characteristics										x	x	x	x
Depression, anxiety, perception of social support										x	x	x	x
Gratitude, purpose, perceived and secondary control, wellbeing, character strengths										x	x	x	x
Anthropometric measures										x	x	x	x
HIV risk behaviors and status											x	x	x
Household wealth										x	x	x	x
Intimate partner violence											x	x	x
Alcohol and substance use										x	x	x	x
Sleep quality										x	x	x	x
Pregnancy and children											x	x	x

^a^Enrolment and interventions have already taken place as part of our previous three trials. This diagram reflects the timeline of participants in those studies

^b^Academic grades will be collected as they become available, with grades being collected pre-and post-follow-up visit

#### Sample size {14}

Combining the three RCTs from which participants will be recruited, our expected sample size will come to ~ 720 youths with elevated symptoms of anxiety or depression at baseline (see inclusion criteria). However, since this is a long-term follow up study, based on our previous experience working in this setting [20], we believe that we will have an attrition rate of ~ 40%. As such, our target sample size for main analyses in this follow-up study is expected to be 432 participants. Of note, this sample size leaves us sufficiently powered: for the most complex planned analyses (multi-level models with four repeated measures), we could detect a small-medium effect size [28] with a power of .8 and a p-value (alpha level) of .05 [67].

#### Recruitment {15}

The study team members will re-establish contact with the high schools which participated in Shamiri’s previous RCTs. School staff will then trace the contacts of the participants in the previous studies by identifying their current class (for those who haven’t graduated, i.e., those who were in form 1 at the time of intervention) or contact information such as phone number (for those who were in forms 2–4 at the time of intervention and have now graduated). Research assistants on the study team will be trained to collect informed consent and to enroll participants in this study. Participants will be notified about the study by the research assistants on our study team. As part of this process, participants will be informed that participation is voluntary and that they can opt out at any time. They will also be given an opportunity to ask questions, after which they will be asked to give informed consent/assent. As compensation for participation, adult participants no longer in school will receive Kenyan Shillings (KES) 500 (USD 5), and an additional KES 300 (USD 3) if they opt to complete additional health measures with the use of a smart watch. Child participants and those in school will be compensated for their participation in accordance with the wishes of school staff at their high school. In past trials, students have been compensated with small prizes such as t-shirts, notebooks, pens, and water bottles.

### Assignment of interventions: allocation

#### Sequence generation {16a}

All assignment to interventions has already been completed, and no new randomization will be necessary for this present protocol. In the first RCT from which participants will be drawn [15], participants were randomized using a randomization script in R (i.e., using computer generated random numbers), and were not stratified in any way. In the second RCT from which participants will be contacted [20], participants were randomized using a randomization script in R and were stratified by sex and by form (i.e., school grade). In the final RCT from which participants will be drawn [21], participants were randomized using an R script offsite by a third party, and randomization was stratified by sex and form and checked for balance on age, GAD, and PHQ baseline scores.

#### Concealment mechanism {16b}

All students included in these past three studies from which participants will be contacted [15, 20, 21] were randomized at one time per study using a computer-generated randomization sequence; thus, the study team had no way of knowing the allocation sequence prior to assigning participants to interventions and had no control over which participants were assigned to which intervention or control conditions.

#### Implementation {16c}

Participants from all three past RCTs included in this study were enrolled by members of the study team based in Kenya. In the third and largest RCT [21], an offsite researcher generated the allocation sequence in order to assign all participants to interventions. In the first two RCTs [15, 20], an onsite member of the study team generated the allocation sequence that was used to randomly assign participants to study conditions.

### Assignment of interventions: blinding

#### Who will be blinded {17a}

In all three past trials from which participants will be drawn, participants, lay-providers, and study staff working with participants could not stay blind to condition, however, they were kept blind to study hypotheses. Lay-providers and participants were not informed which groups were expected to be most effective and were told that the studies were intended to test different programs to improve wellbeing and academic performance. For the current trial, study staff collecting measures from participants will be kept blind to participant intervention condition.

#### Procedure for unblinding if needed {17b}

The only circumstance under which unblinding may become necessary is in case of an emergency (e.g., participant risk). If this occurs, the participant’s assessors will be kept blind to their condition, and other study staff and supervisors who are not collecting outcomes will be informed of the participant’s study condition if necessary.

### Data collection and management

#### Plans for assessment and collection of outcomes {18a}

Three-to-four years after participating in a Shamiri RCT, participants will complete a battery of assessments, to include outcomes from previous RCTs, as well as additional behavioral and health assessments. Primary outcomes include symptoms of depression (as measured by the PHQ-8), symptoms of anxiety (as measured by the GAD-7), academic performance (standardized scores representing grades), and personal satisfaction with social support (MSPSS). Secondary outcomes include character strengths (EPOCH), wellbeing (SWEMBS), sense of life purpose (PILS-12), gratitude (GQ-6), sense of primary and secondary control (PCSC, SCSC), quality of sleep (ASWS), alcohol and substance use (AUDIT, ASSIST), pregnancy and children, intimate partner violence, HIV status and risk behavior, HRV (smart watch measurement), physical activity (smart watch measurement), history of hospitalization, and vital and anthropometric measures. Socioeconomic status and demographics will also be collected (Household Wealth and Demographic questionnaires). Study measures, scales, and questionnaires are further detailed under {12}.

Self-reported measures, including primary measures of depression, anxiety, and social support will be collected from participants immediately following the consenting/assenting process. Consent/assent will be collected by a member of the study team, who will be trained to give information to the participants about what will be collected from them. Participants will then visit with a clinician for collection of more sensitive health and behavioral data. The clinician will be trained in and apply standard procedures for administration of all health and behavioral measures. All data will be entered into a tablet or computer upon collection, and each participant will be identified in this database only by their study ID. The study team will promote data quality by training the data collection team on adherence to study protocols and rules of trial conduct, including proper data collection techniques. All those who interact with private participant data will have up-to-date human subjects training certifications from the federal government. We will conduct periodic data entry checks and will use range checks on all continuous data points, as described under {19}.

#### Plans to promote participant retention and complete follow-up {18b}

Study team members will work with school officials to trace the contacts of participants in our previous trials. School officials will identify the current class of participants still in school and the phone numbers of participants who have graduated. The school staff will be compensated for their efforts to trace participants.

To encourage participation in our study, adults will be compensated with KES 500 (USD 5), in addition to reimbursement of travel and refreshments. Minors will be encouraged to participate with an incentive agreed upon with each individual school, as decided by school administrators. For example, one school wishes for student participants to be compensated with a KES 500 (USD 5) supermarket gift card. Among participants who wish to wear a smart watch for 2 weeks to provide additional health measures, adults will be provided with a KES 300 (USD 3) incentive and minors with a reward agreed upon by the staff at their school. Overall, the Shamiri program was well-received and participants were grateful to be involved. We hope to utilize this reciprocity when re-contacting participants.

#### Data management {19}

All follow-up data will be collected with a tablet or computer via KoBoToolbox (KoBo), an open-source data collection software developed by the Harvard Humanitarian Initiative [68]. Once the data is entered, it will reach a server physically located in Massachusetts, USA, where it will only be accessible with the Shamiri KoBo account username and password. The KoBo server administrator could access this data only if we give explicit instructions to do so. KoBo user passwords are stored fully encrypted on the KoBo server, using Django open-source framework. Django uses the PBKDF2 algorithm with a SHA256 hash, which is considered very secure. For additional caution, only participant’s study ID numbers, not participant names, will be stored on KoBo.

Upon recruitment into this study, all participants will be assigned a unique and de-identifying participant ID number. All follow-up data collected as part of this study will be associated with the participant’s unique ID. The database linking unique IDs with identifiable participant data will be stored electronically in an encrypted file using a separate password to protect participant identities. Only select study team members have access to data which identifies participants. Data access will be limited to study staff with IRB approval. Any team member who will conduct data collection or data entry will sign confidentiality agreements before handling data and receive formal training in the cruciality of participant confidentiality. All data points requiring the study team to enter a number will have range limits which have been pre-defined using the KoBo software. Full data entry and storage protocols to protect patient confidentiality are detailed in section {27} below.

#### Confidentiality {27}

We will separate information which identifies participants from sensitive participant data and make only fully de-identified data public. The database containing sensitive data will be associated with a participant ID which has been assigned by study staff. As noted above under {19}, we will use the software called KoBo Collect for data entry, which is fully compliant with the European Union’s General Data Protection Regulation. Once the data collection is complete and reaches the KoBo server, we will download the responses to a password-protected and encrypted database on our Shamiri server and delete them from the KoBo server once analyses are complete.

All study data will be de-identified and stored in a password-protected and encrypted database only accessible to study staff. Select members of the study team will have access to this database via confidential usernames and passwords. All information which identifies participants to sensitive study data will be stored in this password-protected and encrypted database. A list of participant names and corresponding study ID numbers will be stored in a separate, password-protected folder of Shamiri’s password-protected and encrypted electronic database. Any features which could potentially identify participants to their sensitive data will be removed from the aggregate data presented to those outside the study team (e.g., in presentations or manuscripts). The data collection team will know participant names and the information participants choose to share with them. The study clinician, who will collect the most sensitive health, behavior, and life outcome measures, will not have access to participant names or any identifiable data. However, the study clinician has received in-depth human subject protection training and will sign an agreement to adhere to confidentiality procedures applied in healthcare contexts.

#### Plans for collection, laboratory evaluation, and storage of biological specimens for genetic or molecular analysis in this trial/future use {33}

N/A; this trial does not involve collection or analysis of biological specimens.

### Statistical methods

#### Statistical methods for primary and secondary outcomes {20a}

For analyses of measures that were collected in previous studies (e.g., anxiety, depression, social support, and high school academic grades), we will analyze differences in trajectories over time (the whole trajectory, and between specific time points) of these outcomes between the Shamiri intervention and control group from baseline to 3-4 year follow-up. As in previous analyses comparing changes over time in these measures between the intervention and control groups [15, 20, 69, 70], we will use multi-level models with crossed random effects for lay-providers and schools and nested random effects for individuals within groups within schools. A significant effect for condition over time favoring the intervention group will indicate that the intervention group has significantly outperformed the control group. We will also analyze the trajectory and change over time in absolute terms for the intervention group and analyze the differences between conditions at each time point. Finally, we will analyze the standardized effect sizes (calculated as mean gain scores comparing the intervention and control groups) for each measure from baseline to 3-year follow-up. We may test slight variations on the planned models, to assess need for different random effects structures (e.g., inclusion of random slopes in addition to random intercepts for school, groups, and participants). Model selection among these variations will be based on information criterions (the Akaike Information Criterion (AIC) and Bayesian Information Criterion (BIC)), statistical tests, and numerical identifiability of the statistical models.

For analyses of measures that were not collected at pre-intervention (e.g., history of hospitalization, drug, tobacco, and alcohol use, HIV risk, ambulatory measures), we will be unable to include multiple time points. Instead, we will compare these outcomes between the intervention and control groups with hierarchical models using tests of location difference (e.g., *t*-tests) for metric outcomes and logistic models (e.g., odds ratios) for categorical outcomes to test for significant differences in rates and severity of these outcomes in the intervention group compared to the control group. These models will include control and confounding variables as necessary, likely including age, gender, and school with a lower bound at zero.

#### Interim analyses {21b}

No interim analyses are planned while data collection is ongoing. However, because of the large number of study outcomes, results may be reported across more than one manuscript to enable thorough reporting. We do not have study stopping guidelines in place because we do not anticipate that the study will cause harm to participants. If individual participants appear to present with serious risk of harm to self or others, the expert clinicians involved in the study could decide to withdraw the participant from the trial and instead provide or refer to other more intensive or specialized resources.

#### Methods for additional analyses (e.g., subgroup analyses) {20b}

In secondary, exploratory analyses, we will compare long-term outcomes in the growth-only, gratitude-only, and sense of purpose-only interventions to long-term outcomes in the control group and in the Shamiri Intervention group. For these analyses, we will use the same model types as described above but using the corrections for multiple testing to keep the family-wise error rate. Additional analyses (e.g., mediator and moderator analyses, or analyses of baseline data) will be conducted in an exploratory fashion rather than as part of main analyses described in this protocol. Such exploratory analyses will be disseminated in separate publications from main analyses.

#### Methods in analysis to handle protocol non-adherence and any statistical methods to handle missing data {20c}

Prior to analysis, univariate, bivariate, multivariate, distributional, and missing data characteristics of the data will be examined, with subsequent models adjusted (e.g., using robust estimators and inference, appropriate error distributions, non-parametric approaches) to ensure data-analytic assumptions are met. Missing item-level data will be accommodated using full information maximum likelihood or multiple imputation methods if the assumption (missing at random; MAR) for these mitigations are met; in some cases, missing subject-level data will be omitted from analyses because, for many physical health outcomes of interest, we will not have baseline data [71, 72]. The full information maximum likelihood (FIML)/imputation procedures will permit the inclusion of all participants for whom we have at least partially completed outcome assessments. If there is indication of non-ignorable missing data patterns, we plan to gauge the potential bias incurred thus and check the feasibility of statistical approaches (selection models, pattern-mixture models) that can mitigate the bias. All intervention sessions will have already taken place; thus, no procedures are necessary in this present follow-up study to ensure intervention protocol adherence. Those administering measures will be carefully trained and supervised to ensure measurement protocol adherence (see {18a} above).

#### Plans to give access to the full protocol, participant level-data, and statistical code {31c}

The authors plan to grant full public access to study protocol materials and to statistical code for main analyses, as well as access upon request to de-identified participant data.

### Oversight and monitoring

#### Composition of the coordinating center and trial steering committee {5d}

The Shamiri Institute headquarters in Nairobi, Kenya will act as the coordinating center. The headquarters are in a secure, locked building with secure, locked rooms inside of which the study team may safely store data. The AMHRTF headquarters, also a secure building with secure data storage locations, in Nairobi, Kenya, will serve as another study center. The trial steering committee consists of the study PIs (KVC, a doctoral student in clinical psychology and co-founder and scientific director of Shamiri Institute and TO, co-founder and CEO of Shamiri Institute), a number of professors of psychology and psychiatry (JW, EP, DN), a professor of statistics (TR), and the master-level research manager of Shamiri Institute (NJ). Members of the trial steering committee meet approximately weekly to plan and manage the trial and discuss issues as they arise; they also exchange frequent emails. The trial steering committee will plan the trial protocol, oversee collection and entry of data, supervise study staff during protocol implementation, oversee participant recruitment, and review and address potential adverse events and cases of risk as they occur.

#### Composition of the data monitoring committee, its role and reporting structure {21a}

An external, independent Data Safety and Monitoring Board will not be recruited for this trial for several reasons. First, many members of the study team (e.g., EP, JW, CW, DN, VM, CM) have doctoral degrees in clinical or counseling psychology or degrees in psychiatry, and most of these individuals have many years of experience working with Kenyan youths specifically. Relatedly, several on-the-ground supervisors and a primary care clinician on the study team have degrees and clinical experience in psychology or medicine and are qualified to address emergencies and study issues. Second, the intervention was already delivered to all participants, thus, no intervention will be trialed or delivered during this study, thus, there will be no need to stop delivery of the intervention or remove people from it. Therefore, data and participant safety will instead be monitored by the highly qualified team of scientists and practitioners on the study team.

#### Adverse event reporting and harms {22}

There are no interventions being delivered during this trial, so there will be no collection necessary of intervention-related adverse events. In order to abide by local customs and preferences of guardians, participants who are minors or in schools will not be asked directly about suicidality, IPV, HIV risk, or pregnancy and children unless a school explicitly approves these questions for their students; participants who are adults and no longer in high school will be asked about these topics directly. If, during the course of outcome collection, adult participants reveal risk of harm to self or others, current IPV victimization or perpetration, or other high-risk circumstances or behaviors, they will receive risk assessment and management per the emergency protocol (see supplementary materials) and will be referred to appropriate resources identified by local experts on the study team. If minors incidentally (i.e., without being prompted) reveal or say something that suggests potential risk of harm to self or others, current substance abuse, current IPV victimization or perpetration, or other high-risk circumstances or behaviors, they too will receive risk assessment and management. Per the emergency protocol and as outlined in previous literature [73], this risk assessment and management will involve consultation with members of the study team who are professional practitioners with experience working in Kenya (see supplementary materials); however, for adolescents, coordination of referrals must be mediated by guardians (e.g., school officials or parents), instead of provided directly to participants. Additionally, cases identified as high risk on which IRB consultation is necessary and any adverse events occurring during the study implementation will be reported to the IRB of record.

#### Frequency and plans for auditing trial conduct {23}

This trial does not involve intervention delivery, and the study procedures are highly unlikely to result in adverse events; thus, the trial will be monitored only by the study team, by the IRB of record (Kenyatta University Ethical Review Committee), and by the trial sponsor, the Templeton World Charity Foundation, all of which will require frequent updates on trial proceedings. If any of these parties have concerns about trial conduct, the study team may ask an independent auditor to help identify and resolve these concerns.

#### Plans for communicating important protocol amendments to relevant parties (e.g., trial participants, ethical committees) {25}

Protocol amendments will be communicated first to the study team through email or routine meetings. Then, as the investigators judge it necessary, these amendments will be communicated to the relevant parties such as the IRB of record (Kenyatta University Ethical Review Committee), the trial sponsor, and the public via the study’s Pan-African Clinical Trial Registry (PACTR) registration page. Additionally, if the IRB and study team deem it appropriate, the participants may be notified of protocol changes via phone, email, and/or school announcements.

#### Dissemination plans {31a}

Findings of this study will be disseminated through peer-reviewed journal articles, conference presentations, and sharing of code, protocols, and potentially manuscripts and data on open-access platforms such as Open Science Foundation (OSF). The authors also plan to share findings in popular media outlets when possible (e.g., through blogs, podcasts, and online articles). The authors aim to make all published materials open access. Reporting of trial results will occur across several articles because of the many outcomes of interest in this study and the secondary and exploratory analyses that may be completed with the data collected.

## Discussion

This article includes a thorough description of the design of an RCT evaluating the long-term health effects of a brief, positively focused school-based mental health intervention delivered by peer lay-providers. Through this study, we will assess whether this intervention produces a long-term effect, over and above that of an active control group, on depressive symptoms, anxiety symptoms, perception of social support, and academic performance. We will also assess the effects of this intervention on a wide range of secondary health and behavioral outcomes, such as activity and heart rate variability, overall physical health, and substance use. This will be the first study to test the effects of character strength interventions on such a rich range of health outcomes over such an extended period.

Previous studies of the Shamiri intervention have shown promise for improvement of adolescent depression and anxiety [15, 20], and now, we would like to investigate if these effects hold over the long-term. The knowledge garnered from this trial (i.e., which health and behavioral outcomes are influenced by Shamiri) will directly inform efforts to disseminate this and other simple, low-cost, and stigma-free mental health interventions in low-resource settings.

The results from this study will add to the evidence base for character strength interventions. If we find that the Shamiri intervention impacts some of these health, academic and wellbeing outcomes in the long-term (3–4 years later), that may indicate that character strength interventions could provide a low-cost, stigma-free, and scalable solution to improve long-term health and behavioral outcomes of youth and adults in low-resource settings. This evidence may be of interest to policy makers, adolescent mental health practitioners, educators, and non-governmental organizations aiming to provide effective, scalable, and culturally appropriate care for young people in low-resource settings.

The trial’s results are subject to consideration of some scientific and practical limitations. First, the naturalistic setting of the schools which many of our participants attend leave us with less control over the study setting. As a result, there may be certain threats to internal validity. For instance, the time point of measure is likely to differ between the three RCT cohorts we will contact for follow-up. For reasons out of our control, such as the Kenyan general election cycle and KCSE examination period at schools, we will take follow-up measures between 3- and 4-years post-intervention. It is our position that threats such as these are counterbalanced by external validity provided by our naturalistic setting [74]. Second, contacting participants 3–4 years later may result in a biased sample of participants, as we may be more able to re-contact certain participants for follow-up. For instance, those participants that are still in school may be easier to reach through the school than those that have already graduated. Finally, certain health and behavioral measures, such as HIV status and pregnancy, are not considered appropriate for youths who are still in school, and this will limit our sample size for these analyses. Our study team has conducted three RCTs in a similar setting in Kenya and will apply best practices learned from our previous work [75].

Future research will be needed to identify additional low-cost, scalable, and stigma-free solutions to target mental disorders among youth in low-resource settings. For example, future research could collect long-term follow-up measures of participants in Shamiri digital, a single-session remote intervention which showed promising reductions in depressive symptoms over the long-term [69]. This study could also compare the long-term effects of Shamiri digital to digital cognitive behavioral therapy [76].

Our multicultural and multidisciplinary team is aware that a multitude of efforts will be needed to address global mental health challenges. We hope that this trial will provide a well-powered test of the effects of brief, lay-provider-delivered, positively focused interventions on a variety of health and wellbeing outcomes, thus informing research, policy, and practice in Kenya and potentially beyond.

## Trial status

Protocol version 1.0 (January 12, 2022)

Recruitment has not yet started, and we anticipate completion of recruitment on approximately October 26, 2024.

## Supplementary Information

**Additional file 1.**

**Additional file 2.**

## Data Availability

A de-identified dataset used for the analyses of the effects of the intervention on primary and secondary outcomes will be made publicly available upon request from the authors once the trial is concluded and all data has been analyzed as planned by the authors. This dataset will remain available at least three years following the conclusion of the study.

## Abbreviations

WHO: World Health Organization
LMICs: Low-and middle-income countries
SSA: Sub-Saharan Africa
RCT: Randomized controlled trial
AMHRTF: African Mental Health Research and Training Foundation
GAD: Generalized Anxiety Disorder Screener
PHQ-8: Patient Health Questionnaire
IRB: Institutional Review Board
KCSE: Kenyan Certificate of Secondary Education
MSPSS: Multidimensional Scale of Perceived Social Support
SWEMBS: Short Warwick-Edinburgh Mental Wellbeing Scale
WEMBS: Warwick-Edinburgh Mental Wellbeing Scale
EPOCH: Engagement, Perseverance, Optimism, Connectedness, Happiness Measure of Adolescent Wellbeing
PILS: Purpose in Life Scale
GQ-6: Gratitude Questionnaire-6
PCSC: Perceived Control Scale for Children
SCSC: Secondary Control Scale for Children
ASWS: Adolescent Sleep-Wake Scale
AUDIT: Alcohol Use Disorders Identification Test
ASSIST: Alcohol, Smoking and Substance Involvement Screening Test
VAWI: Violence Against Women Instrument
HRV: Heart rate variability
BMI: Body mass index
KES: Kenyan Shillings
KoBo: KoBo Toolbox
AIC: Akaike Information Criterion
BIC: Bayesian Information Criterion
MAR: Missing at random
FIML: Full information maximum likelihood
PACTR: Pan-African Clinical Trial Registry
OSF: Open Science Foundation
