# Supplementary material for: Long-term health outcomes of adolescent character strength interventions: 3- to 4-year outcomes of three randomized controlled trials of the Shamiri program
Source: Trials. 2022 May 25;23:443. doi: 10.1186/s13063-022-06394-7 (PMC9132569; doi:10.1186/s13063-022-06394-7)
Supplement: Supplementary file 1 — Additional file 1. [file 13063_2022_6394_MOESM1_ESM.docx]

**Emergency Protocol: Study Team**

This document provides a protocol for assessing student risk when you are worried about a student in the study

Be respectful of the student’s privacy by not saying or doing anything to call unnecessary attention to the situation. Try to have a natural and sensitive conversation, and ask questions in addition to those on this sheet as appropriate to explore students’ feelings. This is a guide including all the essential elements of a risk assessment, but every conversation goes differently, so you may add some things or do things in a different order – that is fine.

1. Explain that you’re going to ask some questions about how the student is doing, so you can better understand and help.
2. First, ask the student generally what has been bothering them, or what this tough time has been like for them. Listen and ask clarifying questions as appropriate.
3. At some point in your conversation, you MUST assess risk of harm to self or others. Get answers to the set of questions detailed here:

**Risk Assessment:**

1. In the last month, have you wished you were dead or wished you could go to sleep and never wake up?
2. In the last month, have you had any thoughts of killing or hurting yourself or someone else?

If no to both, END RISK ASSESSMENT. Student is designated NO RISK. Finish up talking with the student. Report the conversation to Brenda within a week.

If yes to 2, get answers to 3, 4, and 5 below.

If yes to 1 and no to 2, skip to 5 below.

1. Have you thought about how you might do this? For example, what you would use or where you would do it?
2. Do you have any intention of acting on your thoughts?

[If the student mentioned a plan: Do you have any intention of carrying out this plan?]

1. Have you ever done anything, started to do anything, or prepared to do anything to end your life?

[Or, if the student mentioned thoughts of hurting someone else: Have you ever done anything, started to do anything, or prepared to do anything to end that person’s life?]

1. If yes to 5: when did you do that, and what did you do exactly?

**No Risk**

If no to questions 1 and 2, then END RISK ASSESSMENT. Student is designated NO RISK. Finish up talking with the student. Report the conversation to Brenda within a week (e.g., in a supervision meeting).

**Low Risk**

If no to 3, 4, and 5, the student is currently at LOW RISK.

- Thank the student for telling you about this. Tell them you’re glad they told you.
- Tell the student that you’ll ask your supervisor, who is an expert in helping people feel better who are in situations like this, for their advice this week, and come back with a plan for the student next week.
- Tell the student you look forward to talking with them next week, and that you’ll be waiting for them after the group meeting finishes.
- Ask the student to please promise you that they won’t hurt themselves this week, and that they’ll talk with you next week.

After the student leaves, call Brenda immediately to explain what happened. If you need to take any immediate action, she will tell you. Otherwise, you’ll discuss this issue in weekly debrief and will have a path forward before the next week.

**Medium Risk**

If yes to 3, and no to 4 and 5,

OR if yes to 5, but no the 3 and 4, and they say they attempted **more than 6 months ago**,

the student is currently at MEDIUM RISK. Do not leave the student until Brenda has spoken with the student and decided if this student needs to speak to Dr. Wasanga.

- Thank the student for telling you about this. Tell them you’re glad they told you.
- Tell the student that you care about their safety and wellbeing and you’re a bit worried for them, and you’re going to call your supervisor now (Brenda, 0794301420), who is part of the Shamiri team and an expert in helping people during tough times like this. Ask the student to meet with Brenda now to figure out a good path forward.

Stay present during the conversation between your supervisor and the student. The supervisor will determine a path forward quickly, and tell you what the plan is. You will likely be asked to talk with the student again in future weeks, to get the student to promise you they won’t hurt themselves in the coming week and will meet you next week, and to ask the student to give you any things that they considered using to harm themselves or someone else. You or another member of the study team may also be asked to connect that student with school administration.

**High Risk**

or 5 If yes to 3, and 4, the student is currently at HIGH RISK. Do not leave the student until Dr. Wasanga has spoken with the student and communicated a plan to you.

- Thank the student for telling you about this. Tell them you’re glad they told you.
- Tell the student that you care about their safety and wellbeing and you’re worried about them, and you’re going to call your supervisor now (Dr. Wasanga, 0721355108), who is part of the Shamiri team and an expert in helping people during tough times like this. Ask the student to talk with the supervisor over the phone now to figure out a good path forward.

Stay present during the conversation between your supervisor and the student. The supervisor will determine a path forward quickly, and tell you what the plan is. You will likely be asked to talk with the student again in future weeks, to get the student to promise you they won’t hurt themselves in the coming week and will meet you next week, and to ask the student to give you any things that they considered using to harm themselves or someone else. You or another member of the study team may also be asked to connect that student with school administration.

Note: In cases that you deem Medium or High Risk, Dr. Wasanga will make a final determination about risk level after further conversation and triangulation with questionnaires and risk and protective factors and proceed as appropriate. These guidelines are built based on those of the American Academy of Pediatrics (Shain, 2016) and the American Academy of Child and Adolescent Psychiatry (Sondheimer, 2010) and adapted to be appropriate for the context of Kenyan high schools.

**References**

Shain, B. (2016). Suicide and Suicide Attempts in Adolescents. Pediatrics (Evanston), 138(1),

E20161420.

Sondheimer, A. (2010). Ethics and Risk Management in Administrative Child and Adolescent

Psychiatry. Child and Adolescent Psychiatric Clinics of North America, 19(1), 115-129.
