# Supplementary material for: Long-term health outcomes of adolescent character strength interventions: 3- to 4-year outcomes of three randomized controlled trials of the Shamiri program
Source: Trials. 2022 May 25;23:443. doi: 10.1186/s13063-022-06394-7 (PMC9132569; doi:10.1186/s13063-022-06394-7)
Supplement: Supplementary file 2 — Additional file 2. [file 13063_2022_6394_MOESM2_ESM.docx]

## Supplement: MODEL INFORMED CONSENT/ASSENT FORM

**INFORMED CONSENT FORM FOR ADULTS**

**The names of those leading this project are:**

Tom L. Osborn, Project Co-Director, affiliated to Shamiri Institute

Ms. Katherine E. Venturo - Conerly, Project Co-Director, Harvard University, and Shamiri Institute

Dr. Christine M. Wasanga, Project Co-Investigator, Kenyatta University

Dr. John R. Weisz, Project Co-Investigator, Harvard University

Dr. Eve Puffer, Project Co-Investigator, Duke University

Dr. David Ndetei, Project Co-Investigator, Africa Mental Health Research and Training Foundation

Dr. Thomas Rusch, Project Co-Investigator, WU Vienna University of Economics and Business

**Title of the Study**

The study is titled: Long-term health outcomes and dissemination strategies for adolescent character strength interventions

**Protocol Number: PKU/2392/E1528**

**Purpose of the Study**

The purpose of the study is to understand the long-term effects on wellbeing, life outcomes, and health of the Shamiri programs designed to improve the wellbeing, academic success, social support, and mental health of high school students in Nairobi. You may remember that you participated in one of these programs around three years ago. This research will help to increase knowledge of ways to serve and improve the health outcomes of adolescents in Kenya.

**Significance of the Study**

The significance of this study is that it will provide evidence for whether brief, positive-psychology focused interventions that are delivered by lay-providers in schools may help improve health, wellbeing, and academic performance in the long-term of adolescents living in sub-Saharan Africa.

About three years ago, you were assigned to a group program called Shamiri that taught study skills or cognitive and behavioral skills and information about psychology. You met in this group for four weeks.

**Procedures to be followed**

Participation in this study will require that I ask you some questions and I also examine you in order to screen you for further questions about your health and wellbeing. We will also seek information on your academic performance from your high school. You may be asked to wear a watch to collect heart rate and activity data. I will record the information you provide in a questionnaire. After the study is completed, identified data will be destroyed.

**Voluntarism**

Participation in this research is completely voluntary, and you can leave the research at any time it will not be held against you. You may ask questions related to the study at any time. You may refuse to respond to any questions, and you may stop an interview or test at any time.

You are free to provide whatever data and participate in whatever procedures you wish, skipping those that you would not like to participate in

**Discomforts and Risks**

Some questions on the questionnaires participants are asked to respond to may make participants uncomfortable or may ask for information which participants do not wish to share. If that is the case, you may refuse to answer the question or choose to leave the study.

Another risk is the risk of loss of time. You will spend several hours over a day participating in this study. Some participants may need to travel to the Nairobi area to participate, though they will be reimbursed for travel costs.

Additionally, there is some risk that participants may learn things about their health and wellbeing that make them feel upset or uncomfortable, however, participants will be asked whether they would like to participate in each medical examination (blood pressure and BMI tests, HIV test, and using wearable watches) and will only participate in each measure if they so choose. You should feel free to ask the study team and nurse any questions you wish. Finally, participants are free to drop out of the study if they are feeling uncomfortable.

**Benefits**

If you participate in this study, you may be able to help in the expansion of the program's testing in Kenya and elsewhere to improve adolescent wellness, health, and academic success. You will also benefit from compensation (KES 500 for an assessment plus reimbursement for travel, food, and lodging costs if applicable). In addition, you will learn important information about your health and wellbeing. If you elect to provide additional physical measures using a smart watch, you will be compensated with an additional KES 300, and receive additional data about your health such as your physical activity and stress level over the study period.

**Reward**

You will be paid KES 500 for your participation in a questionnaire assessment, a stress task, non-invasive physical measurements including weight, height, blood pressure, hip and waist. You will be given an additional KES 300 should you choose to wear a smart watch for two weeks following this in-person visit. Participants may also receive for free, if they elect, information about their general health and an HIV test with pre- and post-test consultation from a local clinic. This information may be useful to you in maintaining your wellness.

**Confidentiality**

No information that reveals your identity will be released or published without your

consent. The discussions and interviews will be conducted in a private setting. Your name will not be recorded on your questionnaires. The questionnaires will be kept in secure, encrypted online databases for safe keeping. Everything will be kept private and only shared with the study team.

**Contact Information**

If you have questions about the study, call the Shamiri Institute, [Office Phone #], or speak with a member of the study team in person.

However, if you have questions about your rights as a study participant: You may contact Kenyatta University Ethical Review Committee Secretariat on [chairman.kuerc@ku.ac.ke,](mailto:chairman.kuerc@ku.ac.ke)

**Participant’s statement**

The above information regarding my participation in the study is clear to me. The study has been explained to me and I have been given a chance to ask questions and my questions have been answered to my satisfaction. My participation in this study is voluntary. I understand that my records will be kept private and that I can leave the study at any time

Name of Participant: ……………………………………………………………………………………

Signature or Thumbprint Date

Name of Representative/Witness (where necessary) Relationship to Subject

**Investigators statement**

I, the undersigned, have explained to the volunteer in a language s/he understands, the

procedures to be followed in the study and the risks and benefits involved

Name of Interviewer ..........................................................................................

Signature Date

**INFORMED ASSENT FORM FOR CHILDREN**

**Project Title:** Long-term health outcomes and dissemination strategies for adolescent character strength interventions

**Protocol Number: PKU/2392/E1528**

**The names of the Research Investigators leading this project are:**

Mr. Tom L. Osborn, Project Co-Director, affiliated to Shamiri Institute

Ms. Katherine E. Venturo - Conerly, Project Co-Director, Harvard University, and Shamiri Institute

Dr. Christine M. Wasanga, Project Co-Investigator, Kenyatta University

Dr. John R. Weisz, Project Co-Investigator, Harvard University

Dr. Eve Puffer, Project Co-Investigator, Duke University

Dr. David Ndetei, Project Co-Investigator, Africa Mental Health Research and Training Foundation

Dr. Thomas Rusch, Project Co-Investigator, WU Vienna University of Economics and Business

The investigators named above are doing a research project.

**These are the things we want you to know about research project:**

We are asking you to be in a research study. Research is a way to test new ideas. Research helps us learn new things.

Whether or not to be in this research is your choice. You can say Yes or No. Whatever you decide is OK.

**What is the study about?**

This study is being conducted to provide evidence for whether brief, positive-psychology focused interventions that are delivered by lay-providers in schools may help improve health, wellbeing, and academic performance in the long-term of adolescents living in sub-Saharan Africa.

**Why am I being asked to be in this research study?**

The purpose of the study is to understand the long-term effects on wellbeing, life outcomes, and health of the Shamiri programs designed to improve the wellbeing, academic success, social support, and mental health of high school students in Nairobi. You may remember that you participated in one of these programs around three years ago. This research will help to increase knowledge of ways to serve and improve the health outcomes of adolescents in Kenya. Since we cannot contact all the adolescents in Kenya, you are representing many other students who we will not be able to reach.

**What will happen during this study?**

About three years ago, you were assigned to a group program called Shamiri that taught study skills or cognitive and behavioral skills and information about psychology. You met in this group for four weeks. We are contacting you now to meet with a nurse in person to complete further questionnaires about your health and wellbeing and to collect some health information. We will also seek information on your academic performance from your high school. You are free to provide whatever data and participate in whatever procedures you wish, skipping those that you would not like to participate in. After the study is completed, identified data will be destroyed.

**Will the study hurt/risks?**

It is extremely unlikely that participating in this study will hurt you or put you at risk. That said, some questions on the questionnaires participants are asked to respond to may make participants uncomfortable or may ask for information which participants do not wish to share. If that is the case, you may refuse to answer the question or choose to leave the study.

Another risk is the risk of loss of time. You will spend several hours over a day participating in this study. Some participants may need to travel to the Nairobi area to participate, though they will be reimbursed for all travel costs. We expect that the benefits to participant wellbeing and the contribution to scientific knowledge about the effects of character-strength-focused programs for Kenyan adolescents may outweigh this risk.

Additionally, there is some risk that participants may learn things about their health and wellbeing that make them feel upset or uncomfortable, however, participants will be asked whether they would like to participate in each medical examination (blood pressure and BMI tests, HIV test, and using wearable watches) and will only participate in each if they so choose. You should feel free to ask the study team and nurse any questions you wish. Finally, participants are free to drop out of the study if they are feeling uncomfortable.

**What else should I know about the study?**

If you feel sick or afraid that something is wrong with you, speak to a member of the study team who will know what to do. You do not have to answer any questions that are asked of you.

**What are the good things /benefits that might happen?**

You will be paid KES [amount to be determined in collaboration with participating schools and communities] for your participation. This information may be useful to you in maintaining your wellness. If you choose to participate in collection of physical data while wearing a smart watch, you will be paid KES [amount to be determined in collaboration with participating schools and communities].

While we cannot guarantee benefits to participants other than compensation, you may benefit from participating in our study in several different ways. First, you may learn important information about your health and wellbeing. Additionally, if successful, the program tested could be used more broadly in Kenya and elsewhere to improve adolescent wellness, health, and academic success.

**What if I don’t want to be in this study?**

Participation in this research is voluntary, and you can leave the research at any time it will not be held against you. You may ask questions related to the study at any time. You may refuse to respond to any questions, and you may stop an interview at any time. Who should I ask if I have any questions?

If you have questions about the study, call the Shamiri Institute, [Office Phone #], or speak with a member of the study team in person.

However, if you have questions about your rights as a study participant: You may contact Kenyatta University Ethical Review Committee Secretariat on [chairman.kuerc@ku.ac.ke,](mailto:chairman.kuerc@ku.ac.ke) or, [secretary.kuerc@ku.ac.ke](mailto:secretary.kuerc@ku.ac.ke)

**Do I have to be in the study?**

No, you do not have to be in the study. Even if you say yes now, you can change your mind later. It is up to you. No one will be mad at you if you don’t want to do this.

**Signatures**

Before deciding if you want to be in the study, ask any questions you have. You can also ask questions during the time you are in the study.

If you sign your name or put a mark below, it means that you agree to take part in this research study.

Your Name (Printed) Age

Your Signature Date
